# Supplementary material for: MetaRibo-Seq measures translation in microbiomes
Source: Nat Commun. 2020 Jun 29;11:3268. doi: 10.1038/s41467-020-17081-z (PMC7324362; doi:10.1038/s41467-020-17081-z)
Supplement: Supplementary file 10 — Supplementary Data 7 [file 41467_2020_17081_MOESM10_ESM.zip › File2/Confidence_High_Taxonomy/309200_out.krona.html]

Javascript must be enabled to view this page.

members
magnitude
magnitudeUnassigned
count
unassigned
taxon
rank

309200\_out

2

2
superkingdom
2

976
phylum
2

class
2
200643

2
order
171549

171552
2
family

2
genus
838

2
species
28131

SRS016746\_contig\_number\_16989SRS142608\_contig\_number\_contig-100\_517.103985
